# The influence of Antarctic subglacial volcanism on the global iron cycle during the Last Glacial Maximum

**DOI:** 10.1038/ncomms15425

**Published:** 2017-06-09

**Authors:** Silvia Frisia, Laura S. Weyrich, John Hellstrom, Andrea Borsato, Nicholas R. Golledge, Alexandre M. Anesio, Petra Bajo, Russell N. Drysdale, Paul C. Augustinus, Camille Rivard, Alan Cooper

**Affiliations:** 1 School of Environmental and Life Sciences, The University of Newcastle, Callaghan, New South Wales 2308, Australia; 2 Australian Centre for Ancient DNA (ACAD), The University of Adelaide, Adelaide, South Australia 5005, Australia; 3 School of Earth Sciences, The University of Melbourne, Parkville, Victoria 3010, Australia; 4 Antarctic Research Centre, Victoria University of Wellington, Wellington 6140, New Zealand; 5 GNS Science, Avalon, Lower Hut 5011, New Zealand; 6 Bristol Glaciology Centre, School of Geographical Sciences, University of Bristol, Bristol BS8 1SS, UK; 7 School of Geography, The University of Melbourne, Parkville, Victoria 3010, Australia; 8 Environnements, Dynamiques et Territoires de la Montagne, UMR CNRS, Université de Savoie-Mont Blanc, 73376 Le Bourget du Lac, France; 9 School of Environment, The University of Auckland, Private Bag, Auckland 92019, New Zealand; 10 European Synchrotron Radiation Facility, 38000 Grenoble, France

## Abstract

Marine sediment records suggest that episodes of major atmospheric CO_2_ drawdown during the last glacial period were linked to iron (Fe) fertilization of subantarctic surface waters. The principal source of this Fe is thought to be dust transported from southern mid-latitude deserts. However, uncertainty exists over contributions to CO_2_ sequestration from complementary Fe sources, such as the Antarctic ice sheet, due to the difficulty of locating and interrogating suitable archives that have the potential to preserve such information. Here we present petrographic, geochemical and microbial DNA evidence preserved in precisely dated subglacial calcites from close to the East Antarctic Ice-Sheet margin, which together suggest that volcanically-induced drainage of Fe-rich waters during the Last Glacial Maximum could have reached the Southern Ocean. Our results support a significant contribution of Antarctic volcanism to subglacial transport and delivery of nutrients with implications on ocean productivity at peak glacial conditions.

## Abstract (web summary)

Contributions of iron sources to Southern Ocean CO_2_ sequestration during the last glacial period remain uncertain. Here, based on the biogeochemical analysis of subglacial calcites, the authors propose Antarctic volcanism, via subglacial drainage of Fe-rich waters, as a key contributor.

The amplitude of atmospheric CO_2_ change from glacial to interglacial periods is difficult to explain by a single mechanism, although during the last glacial maximum (LGM) atmospheric CO_2_ concentration was largely regulated by iron (Fe) fertilization and decreased vertical exchange of waters of the Southern Ocean^1^. The correlation of dust-borne Fe flux with phytoplankton productivity and atmospheric CO_2_ drawdown during the LGM is clear^1^,^2^. However, fertilization experiments indicated that Fe associated with dust particulates is of reduced bioavailability when compared to that associated with organic iron-complexing ligands^3^.

Subglacial environments are an important source of reactive Fe in dissolved, particulate and organic iron-complexing ligand forms prone to hydrological mobilization^4^. Today, subglacially sourced Fe flux from the Antarctic Ice Sheet is estimated to be higher than the labile Fe flux from dust, and travels hundreds of kilometres offshore, thereby helping to sustain primary productivity in the Southern Ocean^4^,^5^,^6^,^7^. Ice sheets contribute potentially bioavailable Fe to the oceans via iceberg transport of Fe (oxyhydr)oxide minerals^8^, as well as through meltwater discharged from subglacial lake drainage^7^,^9^. During glacial maxima, a similar, if not higher contribution from subglacial drainage of meltwaters should be expected from the Antarctic Ice Sheet because a reduced dissipation of elevated geothermal heat due to ice-sheet thickening would favour basal ice melt^10^,^11^. The region around the margins of the Ross Sea is characterized by high geothermal heat fluxes associated with extensional tectonics^10^,^12^ and local volcanism^13^. This is represented by a suite of 24 tephra spanning in age from 15,370±150 to 670±7 years BP preserved in the Talos Dome ice core, 17 of which are geochemically linked to volcanic eruptions in the Mount Melbourne province in Northern Victoria Land (NVL)^14^. Thus, the sector of NVL near the Ross Sea rift is ideal for exploring whether or not volcanically influenced subglacial processes contributed potentially bioavailable Fe to the Southern Ocean in the past.

Evidence for LGM subglacial processes involved in Fe delivery to the Southern Ocean from the Antarctic Ice Sheets has been elusive until now. Datable material capable of recording the chemical, microbial and hydrological signatures of subglacial environments is required, and previous approaches that involved drilling subglacial lakes and sediments suffered from a lack of precise age control^15^. Subglacial carbonates found in Boggs Valley (71°55'S; 161°31'E; elevation 1,160 m a.s.l., NVL), (Fig. 1) provide the first radiometrically dated petrographic, geochemical and genomic evidence of thermogenic, subglacial drainage events that potentially delivered Fe and other nutrients to the ice margin in the LGM. Boggs Valley is ideally located in an area that is characterized by volcanic complexes, the ages of which span more than 400 Ma^16^, in a region characterized by elevated geothermal heat flux.

The presence of subglacial carbonates in an area confirms the existence of steady liquid water production at the ice-bedrock interface^17^,^18^. Variations in crystal morphology and chemistry in successive growth layers record changes in hydrology, solute concentration, particulate and microorganism load within the basal fluid^19^. This environmental information can be placed into an absolute chronologic framework because the carbonates can be dated precisely with uranium-series methods^20^,^21^. The Boggs Valley calcite data sets suggest a relatively important (but as yet neglected) potential contribution of Antarctic volcanism in inducing basal ice melting and sustaining subglacial discharge at peak glacial conditions. Particulate and solutes released via bio-weathering, in a subglacial aqueous environment supporting microbial communities, could have reached the ice margin and supplied potentially bioavailable Fe to the Southern Ocean. This would explain some discrepancies observed between dust-borne Fe fluxes and ocean productivity in the LGM.

## Results

### Ages and sedimentology

**Figure 1:**
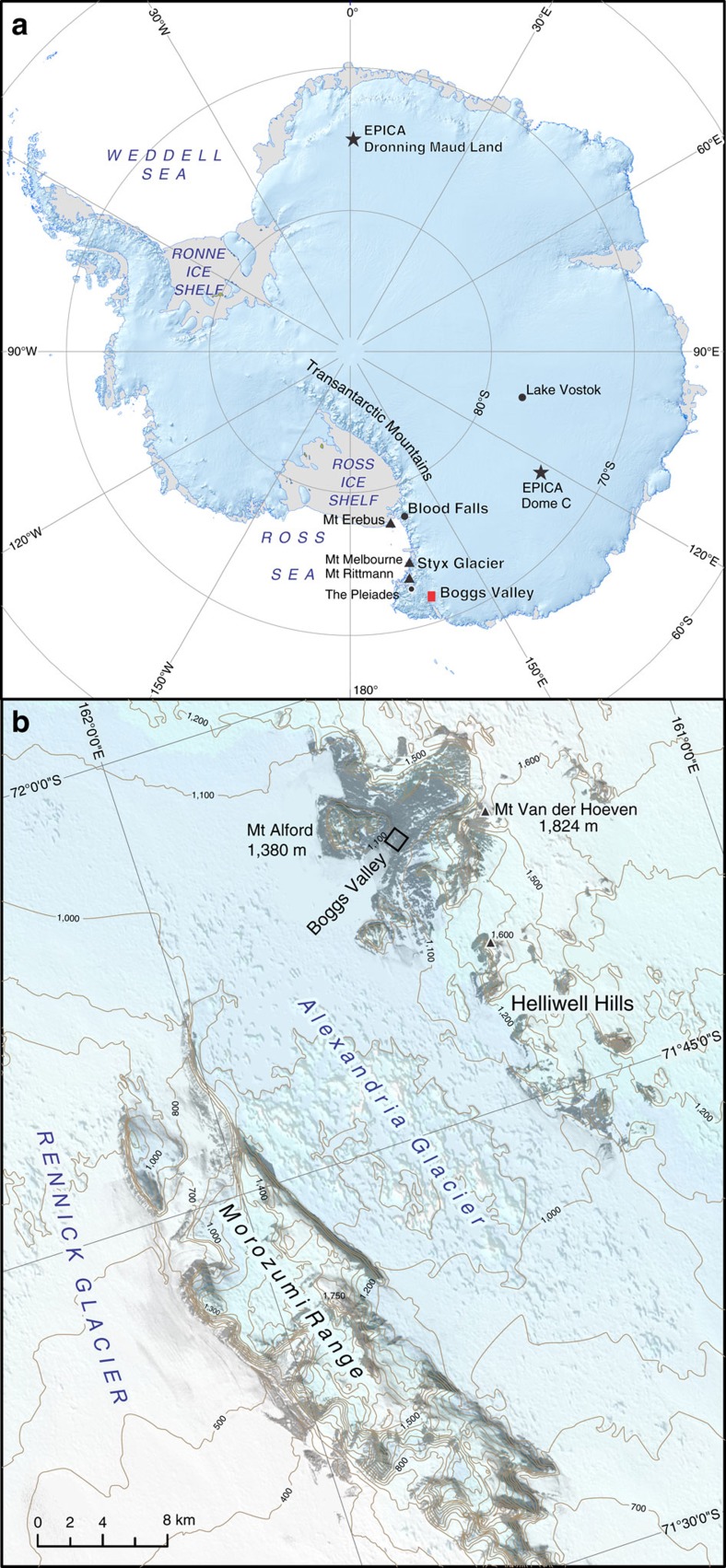
Boggs Valley location, sampling area and geomorphologic context. (**a**) Map of Antarctica with location of Boggs Valley and other sites mentioned in the text; Talos Dome is located circa 100 km south of the sampling site. (**b**) Map created with ASTER GDEM data (by METI and NASA), showing contours. These highlight that Boggs Valley connects the Ice-Sheet plateau with the Alexandria Glacier, whose surface has a lower elevation relative to the valley floor. The Alexandria merges with the Rennick, one of the largest outlet glaciers in NVL. The open square marks the area where subglacial calcite samples were collected from glacially polished rock surfaces.

**Figure 2:**
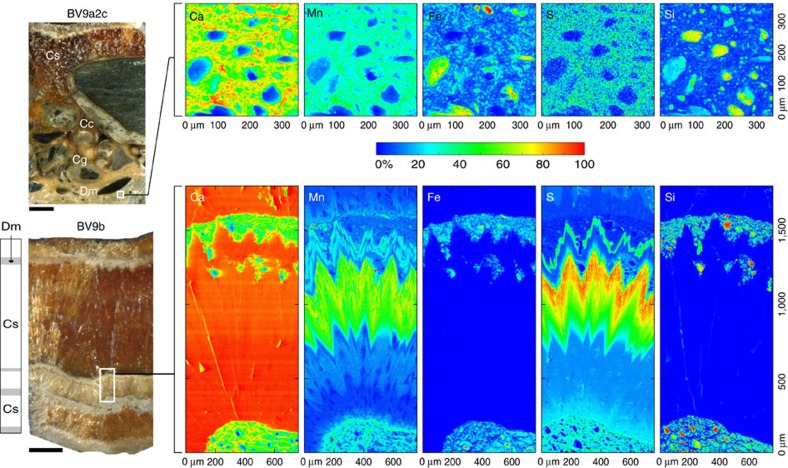
Boggs Valley subglacial calcites petrography and elemental concentration. Sample BV9a2c is representative of deposits found in furrows consisting of rounded clasts cemented by isopachous calcite rims (Cc), granules coated by micrite (Cg: coated grains) capped by columnar Cs. Synchrotron micro XRF elemental maps for the area designated by a white box highlight that maximum concentration of S (as sulfate) is in the calcite matrix. Fe reaches up to 22,000 p.p.m. (as determined by laser ablation (LA)-ICP-MS) in particulate, and it is likely present in the form of oxy-hydroxides when not associated with Si-rich particles. The polished thick section of BV9b is typical of Boggs Valley calcites consisting predominantly of Cs, interrupted by thin layers of Dm, as shown in the microstratigraphic log to the left. The elemental maps for the area designated by a white box show S concentration increasing toward the contact with the overlying Dm. Mn distribution is similar to S and can be interpreted as reflecting a common source or an increase in temperature, as Mn incorporation in calcite is temperature dependent^59^. In the clean Cs, Fe concentration is below 200 p.p.m., with Fe distribution appearing blue on the colour scale. Note that S and Mn concentration distributions follow crystal terminations, providing robust evidence for preservation of environmental signals in Boggs Valley calcites. Scale bar for both BV9a2c and BV9b polished sections is 5 mm. In all maps, elements are reported in normalized counts ranging from high (red) to low (blue) relative concentration according to the 0–100% scale bar.

The U/Th ages of subglacial calcites are expressed in thousands of years (ka) before the year 2000 (b2k). Boggs Valley translucent columnar calcite sparite (Cs) crusts yielded ages from 26.951±0.824 to 17.054±0.332 ka, and their high U concentration (15–56 p.p.m., Supplementary Table 1) suggests that they formed in anoxic/suboxic aqueous pools in the presence of organic compounds^22^. The calcites occur over an area of 1,000 m^2^ of ice-free metamorphic, non-carbonate bedrock (Fig. 1). Their vicinity to moraines and their internal stratigraphy (Fig. 2) are typical of subglacial calcareous deposits found elsewhere and reflect changes in hydrology at the ice/rock interface^17^,^21^.

Cs grew in anoxic/suboxic pockets kept filled by steady production of basal ice meltwaters^17^. Dirty microsparite (Dm) veils interrupt the growth of Cs and can be traced across samples. Notably, a 1–2 mm thick Cs layer bounded by Dm in BV9a2a and BV9b correlates with a Dm veil in BV9a2 and with sediment consolidated by isopachous (phreatic) calcite cement (Cc) in BV9a2c (Supplementary Fig. 1A). Stratigraphic principles suggest these facies are genetically linked and record synchronous episodes of injection of particulate-rich waters into the pockets where clean sparite precipitated. The ages of the Cs bounded by Dm (Cs+Dm facies in sample BV9a2a, Supplementary Table 1) range from 25.135±0.537 ka to at least 23.524±0.446 ka (Dm facies in sample BV9b, Supplementary Table 1). Isopachous cements that indurated sediments consisting of rounded pebbles and granules composed of rocks sourced from up-glacier and deposited in bedrock furrows yielded ages from 22.528±0.661 to 20.891±0.996 ka (Cc fabrics in BV9a2c and BV9a1(i), Supplementary Table 1). As Cc post-dated the deposition of the sediment, it is reasonable to hypothesize that clast cementation eventuated after the precipitation of Dm in BV9b at 23.524±0.446 ka Dm. This suggests synchronous subglacial transport of sediment with pebble-grain size in furrows and particulate injection in basal depressions (Dm). Non-depositional or dissolution gaps interrupting precipitation of Cs mixed with Dm on bedrock protuberances must also be coeval with sediment transport (Supplementary Fig. 1). Thin Dm layers intermittently coat Cs from 21.113±0.283 (BV9a2) to 17.054±0.332 ka, but there is no evidence for chronostratigraphic correlation with indurated sediments (Supplementary Table 1).

### Isotope and chemical properties

The stable isotope composition of Boggs Valley calcites was measured along transects in BV9a2, consisting of Cs, in BV9b, containing both Cs and Dm, and BV11, exclusively formed of Dm. Overall, the δ^13^C_VPDB_ values average −8.0‰ (Supplementary Fig. 2) implying that the dissolved inorganic carbon in the parent waters derived from microbial metabolism^23^ or from subglacial oxidation of organic matter^19^. The δ^18^O_VPDB_ values average −30.3‰ in Dm and −28.5‰ in Cs (Supplementary Fig. 2). These values are similar to those of carbonates precipitated from Antarctic meltwaters^24^.

Facies Cs+Dm formed from 25.135±0.537 to 23.524±0.446 ka are characterized by high sulfur, Fe and manganese concentrations (Fig. 2) and enclose calcium fluoride spherules (Supplementary Fig. 3). Fe, at up to 22,000 p.p.m., is present in its ferric form as (oxyhydr)oxide, most likely ferrihydrite and goethite, associated with particulates (Fig. 2; Supplementary Fig. 4a). Divalent Fe identified by μXANES peaks at 7,126 and 7,130 eV is more common in Cs, where elemental Fe detected by inductively coupled plasma mass spectrometer (ICP-MS) reaches concentrations of ∼100 p.p.m. Sulfur mostly occurs as sulfate (Supplementary Fig. 4b) with concentration up to 3,000 to 5,300 p.p.m. Amino-acid sulfonate was also revealed by μXANES (Supplementary Fig. 4b), suggesting a microbial contribution to the subglacial S pool^25^. Negative-carbon isotope ratio values and sulfonate are consistent with the hypothesis of a subglacial microbial community whose metabolic activity involved organic matter and sulfide oxidation. This is corroborated by ancient DNA sequencing of the subglacial calcites.

### Microorganism associations

Ancient DNA was extracted from calcite Cs and Dm layers under strict ancient DNA protocols. The compact nature of the fabrics ensures that the system was not contaminated by organic compounds or microbes more recent than the LGM. A total of 189 sequences were >97% similar to sequences of known taxa, with the highest proportion in Cs layers pre-dating 21.047±0.521 ka in BV8a and 20.143±0.431 ka in BV9b (Supplementary Data 1, 2, 3). Only 20% could be classified to a genus, and most were observed without clear phylogenetic resolution.

**Figure 3:**
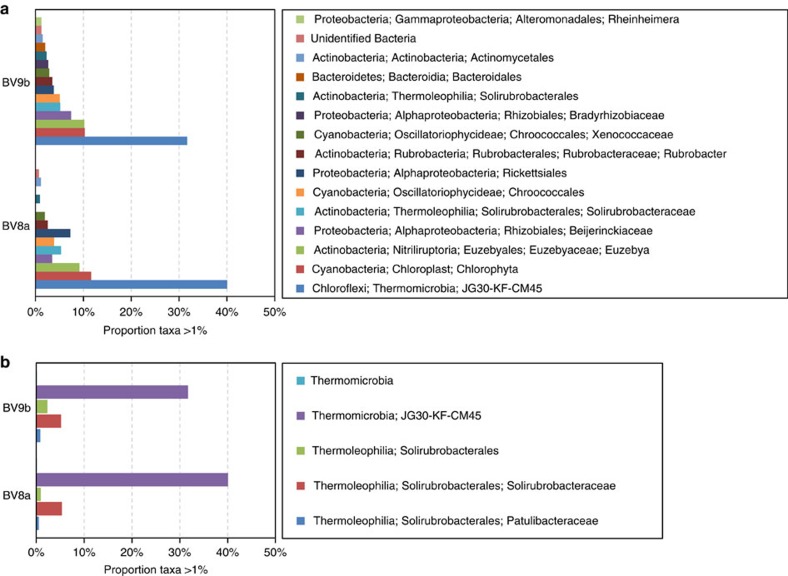
Microbial associations from two sparite samples. (**a**) Bacterial taxa present at >1% of the total diversity graphed along with the lowest taxonomic identification possible. (**b**) Thermophilic bacteria close to known taxa. The proportion of Thermomicrobia (blue) and Thermoleophilia (green) taxa of the total species determined is displayed.

The microbial taxa found in the Boggs Valley subglacial calcites are typical of glaciers, ice, subglacial environments^26^, Antarctic lake sediments and springs^25^,^27^, deep-sea sediments and deep-sea thermal vents^28^,^29^. Some pertain to endolithic communities^30^,^31^ and include the phyla Chloroflexi (40–32%), Actinobacteria (22–25%), Cyanobacteria (18–19%) and Proteobacteria (13–17%). Others are associated to communities with known critical functions for silicate bedrock dissolution, including sulfur reducing and organic matter-oxidising bacteria, while others are known to promote calcite precipitation, including autotrophic, heterotrophic and phototrophic taxa^32^,^33^,^34^ (Fig. 3a). Thermophilic microbial species accounted for 47 and 40% of the DNA sequences determined in clean columnar Cs^35^,^36^,^37^ (Fig. 3b). These thermophilic taxa belong to large phylogenetic groups of microorganisms that thrive at temperatures above 41 °C (refs 38, 39). In contrast to the rich microbial association extracted from Cs crusts (BV8a, BV9b), a glacially deformed, Dm sample (BV11) yielded none of the taxa typical of thermophilic environments. The LGM basal meltwater community in Boggs Valley reconstructed from ancient DNA shares similarities to known Antarctic subglacial environments^5^,^26^, but maintains a unique signal that is indicative of LGM hydrothermal activity in the area.

## Discussion

**Figure 4:**
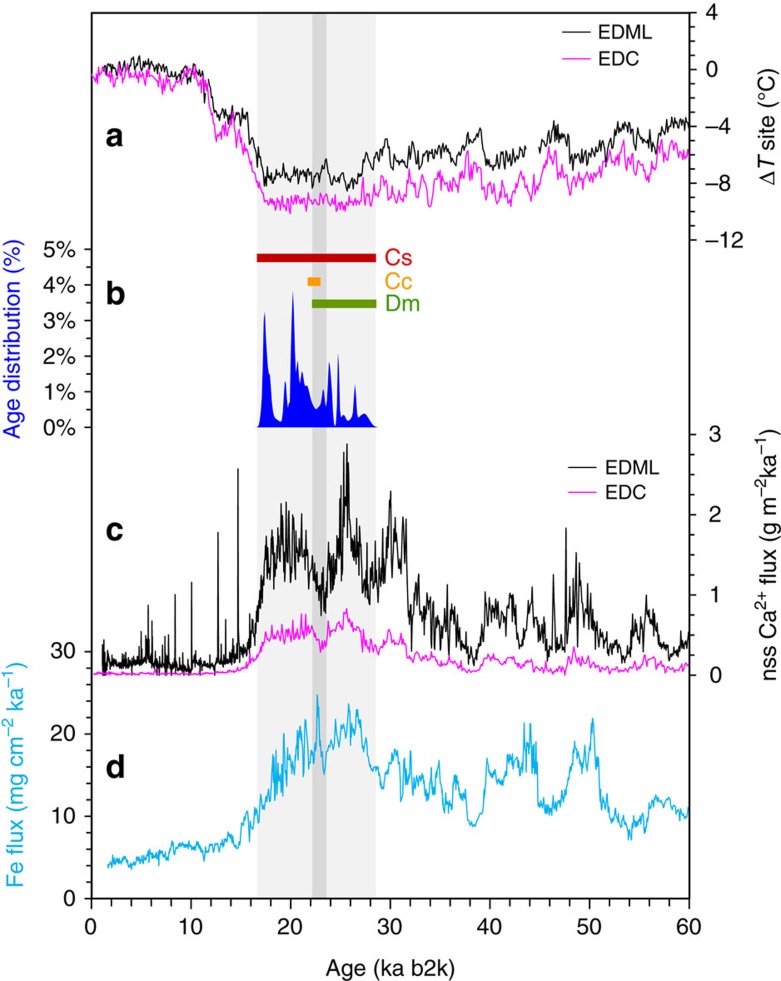
Subglacial calcite age distribution in relation to LGM temperature and dust fluxes. (**a**) EPICA Dome C and EPICA DML temperature anomalies corrected for site elevation^41^; (**b**) the age distribution of Boggs Valley calcites produced by using a normalized sum of each individual Gaussian distribution for all the 39 analyses where the [^230^Th/^232^Th] ratio is >30, including analytical errors (Supplementary Table 1). Analyses where the [^230^Th/^232^Th] ratio is <30 are excluded from the plot. Horizontal bars above the age distribution highlight the changes of fabrics according to ages. Clear columnar Cs spans all ages. Isopachous Cc of clasts (Cc in Fig. 2, Supplementary Table 1) in the indurated sediment has a narrow range of ages, centred soon after the dust minima as identified by non sea-salt Ca fluxes (**c**). Dm is uncommon in layers younger than *ca.* 22 ka.; (**c**) non sea-salt Ca fluxes from EDML and EDC records^71^; (**d**) the total Th normalized Fe flux in the Subantarctic Ocean reconstructed from ODP1090^2^. The dark grey vertical band identifies a discrepancy between Fe flux in ODP1090 and dust fluxes from EDC and EDML records, and coincide with low frequency of subglacial calcite formation. The ages of subglacial calcites correspond to the timing of the LGM (highlighted by the light grey vertical band) in both EDC and EDML, confirming that the calcite precipitates record an accurate archive of maximum ice thickness.

Subglacial calcite growth in Boggs Valley occurred because wet-based subglacial conditions persisted from 26.951±0.824 to 17.054±0.332 ka (Supplementary Table 1), coinciding with the duration of the LGM as recorded in EPICA Dome C (EDC), Talos Dome (TALDICE) and EPICA Dronning Maud Land (EDML) temperature records^40^ (Fig. 4).

For basal meltwater production to be sustained in a polar glacier during glacial maxima, the ice must have been thick enough to limit dispersion of geothermal heat flux to the atmosphere. The glacier thickness (*H*) necessary to sustain basal temperature (*T*_b_) conducive to wet-based conditions during the LGM was modelled by using a thermal conductivity of ice (*K*_ice_) of 2.4 W m^−1^ K^−1^, a mean annual air temperature (*T*_s_) of −29.4 °C, 9.5 °C (ref. 41) below the present temperature of −19.9 °C (ref. 42) and a geothermal heat flux (*G*) of 120.9 mW m^−2^ inferred from magnetic data^10^ or 75 mW m^−2^ hypothesized from modelling^12^:


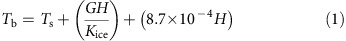


**Figure 5:**
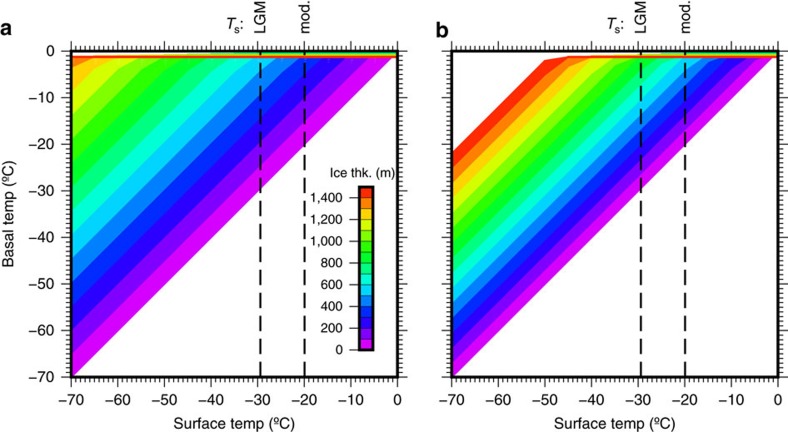
Estimated LGM ice thickness at Boggs Valley. Modelled LGM ice thickness (equation (1)) necessary to induce basal melt for LGM temperature of 9.5 °C lower than present^41^, and geothermal heat flux of (**a**) 120.9 mW m^−2^ and (**b**) 75 mW m^−2^.

Considering the uncertainties in the ice-thickness calculation in equation (1), the LGM glacier that occupied Boggs Valley had to be 580–900 m thick to maintain steady basal melting (Fig. 5). This is consistent with the 800 m estimated for the area obtained from a three-dimensional LGM ice-sheet simulation^43^. This projection suggests that a nominal ice thickness of 750 m was necessary to ensure a hydrous subglacial environment in Boggs Valley (Fig. 5) throughout the LGM.

A high density of measured ages in Boggs Valley calcites (peaks in the age distribution in Fig. 4b) likely coincides with periods when steady basal melting conditions favoured the growth of Cs throughout the LGM (fabric distribution in Fig. 4b). Cs is known to be the product of incomplete re-freezing of basal meltwater when persistent, slow flow fills a subglacial interconnected pore system^17^. In contrast, when measured ages are scarce, it is plausible that Cs growth was interrupted by dissolution, erosion and injection of sediment-laden meltwaters sourced from up-glacier (Supplementary Fig. 1).

A decrease in age distribution occurs at *ca.* 25.1±0.5 and persists until *ca.* 22.5±0.6 ka, which coincides with the ages of isopachous cements Cc. Therefore, the process responsible for the subglacial transport and deposition of coarse grained, rounded sediment and particulates to the subglacial environment of Boggs Valley had declined at *ca.* 23 ka. By considering a cluster of Dm dates around 23.5 ka (23.769±0.296, 23.524±0.446, 23.135±0.537 ka, Supplementary Table 1), it is reasonable to surmise that from 25.5 to 23.5 ka (Fig. 4b), when EDML and EDC record a decrease in dust flux, episodes of discharge of sediment-laden subglacial meltwater with high enough energy to reach the ice margin occurred^7^. Only high-energy current discharged from a subglacial source, such as a lake could have transported pebble-sized sediment sourced from up-glacier into Boggs Valley (Supplementary Fig. 1). Under cold conditions of the LGM in Antarctica, surface melting was unlikely to reach the bedrock through crevasses and sustain subglacial flow^7^. The ancient DNA data suggested that thermophilic microbial taxa were mostly associated with calcites formed during or soon after discharge. This, and the high level of sulfate and fluorine in calcites in layers whose ages cluster around 23.5 ka make plausible the notion that a hydrothermally influenced subglacial lake, similar to Lake Vostok^29^,^44^, discharged into Boggs Valley at, or just before 23.5 ka (Fig. 4b). In addition, the ^13^C depletion recorded in the subglacial calcites indicates that the reservoir was characterized by organic-matter oxidation, or re-mineralization, that resulted in an isotopically light, dissolved inorganic carbon signature. In Antarctica, subglacial lakes show ^13^C depletion^45^, so that it is likely that a subglacial meltwater reservoir was drained following breaching of a hydrological barrier. Pore waters, sustained by steady melting of ice, were flushed through when bursts of discharge waters were injected into the basal interconnected pore system of Boggs Valley.

Transantarctic Mountain (TAM) volcanic activity, from Mount Melbourne to Mount Erebus, to the Pleiades (Fig. 1), is characterized by the release of fluorine, sulfur and Fe-rich fluids^46^,^47^. Critically, the LGM fluoride record in the EDC ice core has been tentatively ascribed to subglacial eruptions in the TAM region^48^. Since geothermal heating in NVL is higher (∼20 mW m^−2^) than in the Lake Vostok region^49^, it is reasonable to hypothesize that a similar deep subglacial lake existed up-glacier from Boggs Valley. Meltwaters released by drainage likely reached the ice-sheet margin by flowing along the axis of the Rennick outlet glacier^9^,^50^,^51^.

We used Icelandic examples^52^ to estimate the volume of ice melt produced by subglacial volcanism for a range of areal extents based on an ice overburden of ∼750 m (Supplementary Fig. 5). The calculated subglacial ice melt was of sufficient volume to offset the overburden pressure exerted by the ice, thereby overcoming a glacio-hydrostatic barrier and generating drainage with adequate energy to keep subglacial conduits open for long periods of time^9^,^50^ and transport pebble-sized particles (Supplementary Fig. 1c)

At *ca.* 17.1±0.3 ka (Fig. 4b) calcite ceased to form because ice-thickness reduction caused dispersion of geothermal heat and a change to entirely cold-based conditions. This age agrees with an early onset of the deglaciation (*ca.* 18 ka) documented in areas of strong ice streaming in East Antarctica^53^,^54^. As a result, the connection between hydrothermally influenced subglacial lakes at the interior of the ice sheet and the margin was severed at the end of the LGM in this sector of NVL.

Under today's global warming scenario, meltwaters discharged from supraglacial environments interacting with subglacial sediments and bedrock, deliver dissolved Fe to the ocean^7^,^55^. In contrast, at peak glacial conditions it was a combination of geothermal heat flux, thick ice and volcanism that sustained subglacial melting. In Boggs Valley, a wet-based subglacial environment hosted organisms using autotrophic and heterotrophic metabolic pathways capable of releasing divalent Fe (Fe^2+^) from the dissolution of silicates and pyrite oxidation^56^,^57^,^58^. This Fe^2+^ may have formed complexes with the natural organic matter in the basal meltwater or remained as dissolved species (<<1 nm), both of which could have been adsorbed onto reactive calcite surfaces. Thus, the amount of Fe^2+^ released can be estimated from the concentration of Fe incorporated in the clean sparite crystals. Using a conservative concentration of 100 p.p.m., the Fe^2+^ dissolved in the LGM basal waters was calculated by extrapolating the experimental distribution coefficient between solid calcite and Fe in solution (D_Fe_^2+^)^59^ at an inferred basal temperature of just above freezing (∼2 °C). The value of D_Fe_^2+^ is 3.6 for precipitation rates of 1.8–6.6 μmol m^−2^ h^−1^ estimated for Cs (whose growth rate is 0.57 μm per year, between 24.634±0.221 and 20.153±0.431 ka in BVa2 and 2.16 μm per year between 20.153 and 17.272±0.226 ka in BV9b). Assuming that the LGM subglacial waters had a Ca^2+^ concentration of 0.5 mmol l^−1^ (ref. 60), the divalent Fe concentration would have been ∼0.32 μmol l^−1^, a value similar to that reported for Antarctic basal ice^61^ and measured in outlet glacier meltwater in Greenland^55^.

The combination of thick ice, geothermal heat flux, volcanism and microbial metabolism very likely resulted in the delivery of bioavailable Fe to the Southern Ocean, and may ultimately have contributed to driving changes in the global C cycle during the LGM. Our new data enables an alternative interpretation of the discrepancies observed in peak Fe flux recorded by ocean sediments at site ODP1090 at *ca.* 23 ka that are not paralleled by an increase in dust flux recorded by ice cores (Fig. 4). In addition to soluble and complexed Fe, the basal hydrological system transported Fe as fine-grained material, including the mineral ferrihydrite, which could have provided a source of potentially bioavailable Fe that reached the productive iceberg-associated sediments^8^. The importance of the new data from Boggs Valley is that they corroborate the hypothesis that subglacial Fe evolved from bio-weathering and was delivered to the ocean by volcanically induced discharge processes in the LGM. Our first finding of thermophilic microorganisms in a well dated archive of subglacial environments and processes support the concept that volcanism had a significant role in sustaining both subglacial hydrology and subglacial microbial communities at peak glacial conditions.

Boggs Valley calcites are not a unique record of volcanic processes in the TAM region. Calcite precipitates found elsewhere have been interpreted as the product of subglacial hydrothermalism^62^, and subglacial eruptions are registered in the sulfate and fluorine records of TALDICE and EDC ice cores^46^. The TAM rift has high levels of eruptive activity related to upwelling and lateral flow of the upper mantle from the Ross Sea Rift^16^. It is also noteworthy that mantle upwelling has been recently associated with waxing and waning of the ice sheets, thereby providing a link between magmatism and glacial cycles^63^. Further research on similar calcites is needed to test the quite plausible hypothesis that Antarctic volcanism, by changing subglacial meltwater discharge of micronutrients, could potentially influence global climate.

## Methods

### Sampling

Samples of brown-reddish subglacial calcite crusts were collected from the bedrock, consisting of glacially polished amphibolite, where these were not covered by snow, within a total area of 100 m^2^ (location shown in Fig. 1b) in the course of a survey targeted to obtain terrestrial cosmogenic nuclide exposure ages on adjacent moraines. The morphology of the crust was typical of similar deposits occurring on exposed carbonate and non-carbonate bedrocks of Alpine glaciers. Thus, the crusts were immediately recognized as potentially bearing information on subglacial processes in Antarctica.

For the present study, the most compact samples characterized by columnar Cs layers (Supplementary Table 1) were selected for dating, chemical and microbial analyses, with the reasonable assumption that the Cs fabrics are compact enough to ensure closed system behaviour relative to trace elements and isotope mobilization. Therefore, all environmental proxy data and ages are likely to reflect the original depositional environment, and are unaffected by post-depositional alteration or contamination following de-glaciation. In addition, dating, chemical and ancient DNA analyses were carried out on sample BV11 exclusively consisting of greenish microsparite matrix embedding angular clasts. Two samples, BV9a1 and BV9a2c consisted of clasts cemented by isopachous calcite. The cleanest isopachous cements (Cc in Supplementary Table 1), which are as thin as 0.1 mm, were sampled by microdrilling following assessment of their primary nature under the optical microscope. Similarly, micrite veils coating clastic grains (Cg in Supplementary Table 1), which resemble a microbial structure were also microdrilled to test if their age was consistent with a subglacial genesis.

### Microscopy and mineralogy

Thin sections of subglacial calcites were observed by optical and epifluorescence microscopy. One millimetre thick wafers of age-equivalent subglacial crusts were polished and ultrasonically cleaned. Imaging at nm-scale resolution on uncoated surfaces was carried out by field emission scanning electron microscopy with a ZEISS Sigma Variable Pressure field emission scanning electron microscopy equipped with Bruker EDS system in backscattered electron mode at the Electron Microscope and X-ray unit of the University of Newcastle, Australia. Semi-quantitative chemical microanalyses were carried out *in situ* on spot areas of *ca.* 500 nm diameter and the normalized weight % calculated with the Quantax software (Bruker nano). Epifluorescence was excited by light at 250–350 nm wavelength and the most common emission band was in the 450–550 nm.

Powder X-ray diffraction on clean calcite and Dm was carried out on a PANalytical h-h diffractometer equipped with a Cu X-ray tube operating at 40 kV and 40 mA. Scans were performed over the range 3–80° with an integrated step size of 0.017° and a counting time of 100 s per step. Identification of minerals, and cell parameter determination were performed using High Score Plus and the ICSD database (PANalytical).

### Dating

U/Th dating was performed on eight Boggs Valley subglacial samples by multi-collector ICP-MS at the School of Earth Sciences, the University of Melbourne, following the analytical method described in Supplementary Table 1. From each sample, powders were drilled from top to bottom in Cs and Dm layers, with density of sub-samples depending on the thickness of the material. Ages were obtained for 50 sub-samples, 7 of which showing 2σ error >±1,000 years (highlighted in red, Supplementary Table 1). In these sub-samples, uncertainties are likely due to detrital Th contamination. In most samples, Dm layers are thinner than 0.1 mm (for example in BV9a2, Supplementary Fig. 1A) and the age had to be reconstructed from that of clean Cs layer below and above. The age of mixed Cs and Dm (Cs+Dm in Supplementary Table 1) in BV9b, is constrained by a *pre-quem* age of 26.307±0.204 ka, and a date for the top Dm precipitated on partially dissolved Cs of 23.524±0.446 ka. It is reasonable to reconstruct, on the basis of correlation with dated Cs+Dm in BV9a2a, an age of *ca.* 25.5 ka (25.135±0.537 ka) for the commencement of Cs+Dm precipitation in BV9b (Supplementary Fig. 1A). Reconstruction of the timing of deposition of sediments was performed by considering that the ages obtained for the Cc (Supplementary Table 1) pre-date the formation of the overlying Cc layer in BVa2c (see Supplementary Fig. 1), but post-date the deposition of the clasts. Given that Cc is as old as 22.528±0.661 ka it is reasonable to infer that transport was coeval with the deposition of Dm in BV9b and BV9a2a and, likely, with the whole-Cs+Dm layer.

The type of age model used to correlate samples is, thus, based on stratigraphic principles, whereby similar, genetically related facies bounded by erosion or non-depositional surfaces (unconformities) or correlative conformities (continuously growing columnar Cs). This implies that the stratigraphy of Boggs Valley subglacial calcites reflects accommodation space available on the substrate (or between substrate and ice) and Cc (+ Dm in BV9a2a and BV9b) formed on topographic highs relative to the lows were sedimentation of clast occurred.

### Geochemistry

Micro X-ray fluorescence (XRF) mapping was carried out at beamline ID21 at the European Synchrotron Radiation Facility (Grenoble, France). Double-polished 300 μm thick wafers were ultrasonically cleaned and loaded in the sample chamber operating at a pressure of 10^−5^ mbar. Maps were acquired by raster scanning with a step of 2 μm in both directions. XRF was stimulated with a monochromatic beam and was collected in the horizontal plane, using a large solid state detector. This geometry, together with the linear polarization of the incident X-ray beam and the vacuum conditions, minimize background signals from elastic and inelastic scattering. The micro-XRF mapping was carried out in an area of 20 × 5 mm to include all microstratigraphic components identified by microscopy. Excitation energy was set at 7.5 keV and 2.6 keV to maximize the XRF yield of the elements of interest and avoid the excitation of Ca, which would easily saturate the XRF detector. XRF spectra were batch-fitted using the PyMca software package.

Micro X-ray Absorption Near Edge Structure (μXANES) investigation was carried out by tuning the exciting energy around the S absorption K-edge (from 2.45 to 2.55 keV) and the Fe K-edge (from 7.1 to 7.2 keV) utilizing a Si(220) double-crystal monochromator and an energy resolution of 0.25 eV (ref. 64). Calibration with internal standards allowed comparison with reference spectra.

For quantitative geochemical analysis we used a Varian 810 quadrupole ICP-MS equipped with helium excimer laser system (School of Earth Sciences, the University of Melbourne). The helium excimer produces an ultraviolet light beam (24 mm × 8 mm) with wavelength of 193 nm and pulse length of 24 ns. Typical scan speed is 20 μm s^−1^. Background measurement and analyses of the NIST612 standard were carried out periodically for drift corrections. Data were calibrated to the NIST612 standard and converted to absolute concentrations using ^43^Ca as internal standard. Background was subtracted and baseline drift corrected by linear interpolation of the standard counts. Because of mass interferences, S and P were quantified by EDS using an internal standard for the regions where these elements were detected by synchrotron-radiation-based micro-XRF.

Stable-isotope analyses were conducted using a GV Instruments GV2003 continuous-flow isotope ratio mass spectrometer at The University of Newcastle, Australia. Samples of ∼0.75 mg were acidified in evacuated septum-capped vials with 0.05 ml of 105% phosphoric acid. The CO_2_ evolved from the reaction was admitted into the ion-source chamber under vacuum in an ultra-high-purity helium gas stream. Sample isotopic ratios were standardized to the VPDB scale using an in-house standard of Carrara Marble (NEW1) and two international reference materials, NBS19 and NBS18. Analytical reproducibility for C and O was better than 0.05 and 0.10‰, respectively.

### Ancient DNA technique

Bacterial amplicon sequencing was undertaken on BV8a, BV9b and BV11a, representative of clear sparite, a sequence of sparite and Dm, and Dm, respectively (Supplementary Fig. 6). Contaminating DNA on the outer surface of the samples was removed by removing ∼2 mm of the outer surface with a Dremel tool, exposing each side to ultraviolet irradiation for 15 min, and soaking in 3% hypochlorite solution, as previously described^65^. DNA was then extracted from the decontaminated microsparite using a modified silica-based protocol developed for low biomass extractions in an ultra-clean, specialized ancient DNA laboratory (The Australian Centre for Ancient DNA at The University of Adelaide)^66^. Sample controls (extraction blank controls) were also processed simultaneously to monitor laboratory and reagent contamination^67^. The 16S ribosomal RNA (rRNA) encoding gene regions were amplified using polymerase chain reaction^68^,^69^. To account for contamination introduced into these low biomass samples via laboratory environment and reagents, sequences identified in extraction blank controls were filtered from the data set^67^,^70^. 16S rRNA libraries were created as previously described^69^ with 38 cycles performed to achieve amplification of limited ancient DNA fragments^65^. 16S rRNA amplicon libraries were then pooled and sequenced on an Illumina MiSeq, generating ∼500,000 reads between the three calcite and one extraction blank control sample. Sequences were then de-multiplexed using CASAVA, trimmed and quality filtered using CutAdapt, and imported into QIIME for downstream analyses. Bacterial species were determined by aligning operational taxonomic units selected by clustering at 97% similarity using UClust to the Greengenes database.

### Data availability

Our DNA data are publically available on the QIITA database: study ID 10766: ‘The Influence of Antarctic Subglacial volcanism on the global Fe cycle during the Last Glacial Maximum'. DNA sample list is supplied in Supplementary Data 1. Processed data are available as Supplementary Data 2 and 3 (OTU tables in BIOM format). Raw data files have also been uploaded to the NCBI SRA database as BioProject ID: PRJNA386567; BioSample IDs: SAMN07139230, SAMN07139231. All other relevant data are available upon request from the authors.

## Additional information

**How to cite this article:** Frisia, S. *et al*. The influence of Antarctic subglacial volcanism on the global iron cycle during the Last Glacial Maximum. *Nat. Commun.* **8,** 15425 doi: 10.1038/ncomms15425 (2017).

**Publisher's note:** Springer Nature remains neutral with regard to jurisdictional claims in published maps and institutional affiliations.

## Supplementary Material

Supplementary Information Supplementary Figures, Supplementary Table and Supplementary References

Supplementary Data 1 list of Boggs Valley subglacial calcite samples analysed for DNA. Sample BV11, consisting of angular clasts cemented by microsparite, is sterile (see text for details.)

Supplementary Data 2 OTU .txt file relative to the DNA data for Boggs Valley calcite samples BV8a and BV9b. BV 11 is sterile.

Supplementary Data 3 OTU data in .Biom format relative to samples BV8a and BV9b.
