# Supplementary material for: The influence of Antarctic subglacial volcanism on the global iron cycle during the Last Glacial Maximum
Source: Nat Commun. 2017 Jun 9;8:15425. doi: 10.1038/ncomms15425 (PMC5472753; doi:10.1038/ncomms15425)
Supplement: Supplementary Information — Supplementary Figures, Supplementary Table and Supplementary References [file ncomms15425-s1.pdf]

Supplementary Figure 1

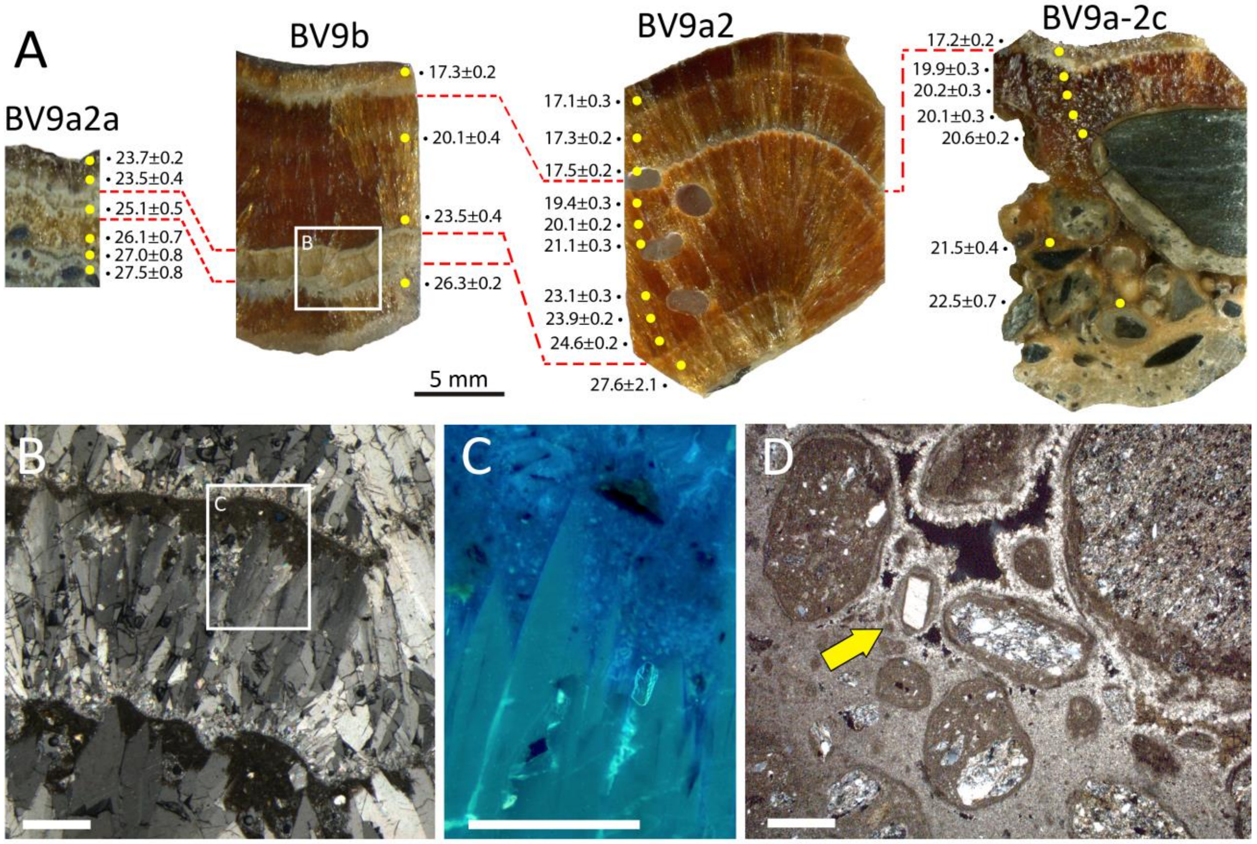

Microstratigraphy and ages of calcite crusts and indurated sediments found in Boggs Valley.

A) Correlative relations between samples. The dashed red lines identify ages of genetically related facies as reconstructed from both radiometric dating and microstratigraphy. Yellow dots highlight locations of samples drilled for U-series dating (Supplementary Table 1, rounded to the first decimal). By considering stratigraphic correlations, and the age distribution in text Figure 4b, it is reasonable to infer that subglacial meltwater transport occurred from ca. 25.5 to 23.5 ka. The velocity of the current that transported the clasts would have been in the range 20 to 50 cm/s for particles 4 to 10 mm in size. Isopachous calcite fringes are typical of a phreatic aqueous environment, with the voids filled by flowing water after sediment deposition. The sedimentary properties of the clasts suggest origin as channel lag deposits. B) Micrograph in cross-polarized light (thin section) of the area highlighted by square in BV9b, which is also illustrated in Figure 2 in the text; C), epifluorescence image of the area detailed by rectangle in B showing Cs at the boundary with Dm. Cs is characterized by emission band at 450 to 550 nm. Similar fluorescence in continental calcites has been ascribed to Fulvic and Humic acids (FA and HA) and Particulate Organic Matter (POM) (<0.7 µm in size)[1]. Elsewhere, dark coloured calcites commonly have twice the concentration of POM, FA and HA than light-coloured ones[1]. Incorporation of the products of microbial decomposition of organic matter in Boggs Valley calcites agrees with the

interpretation of negative $\delta^{13}C$ values as related to microbial metabolism (see main text). The tips of the Cs crystals show dissolution and corrosion, likely related to the injection into the system of understaturated waters. In contrast, luminescence is dispersed in Dm. This suggests that nucleation of Dm occurred on abundant organic compounds and silicate particles transported into the system by exogenous waters. By abating interfacial energy barrier, particulate and organic molecules favoured formation of myriads of small crystals (microsparite)[2]; D) Thin section detail of BV9a2c showing clasts of metamorphic sandstone, amphibolite and gneiss embedded in micrite and/or coated by micrite veils (Cg in Supplementary Table 1). Amphibolite and gneiss are typical of the regional Palaeozoic basement[3], whereas metamorphosed sandstones outcrop above the sampling site, at the crest of the Helliwell Hills, and pertain to the Permo-Triassic Beacon-Supergroup[4]. Angular to sub-rounded amphibolite clasts reflect short lived transport. By contrast, metamorphosed sandstone granules and pebbles are rounded, which suggest transport by aqueous currents from up-glacier. Granules coated by micrite "envelopes" indicate cyanobacteria or endolithic microbes activity[5]. Cyanobacteria can be photosynthetic-independent[6], and the lack of information on bacterial functions in Antarctica limits our ability to reconstruct the specific species and functions of the ancient microbial community preserved in the calcites. However, ages obtained from micrite coatings (Cg in Supplementary Table 1) suggest that they formed in the LGM, thus, they are likely related to sub-glacial bio-mediated precipitation of calcium carbonate crystals. Their morphologies are typical of lacustrine environments[6], and allow an up-glacier provenance to be inferred, likely from subglacial lake discharge. Scale bar in B, C, D = 0.5 mm.

Supplementary Figure 2

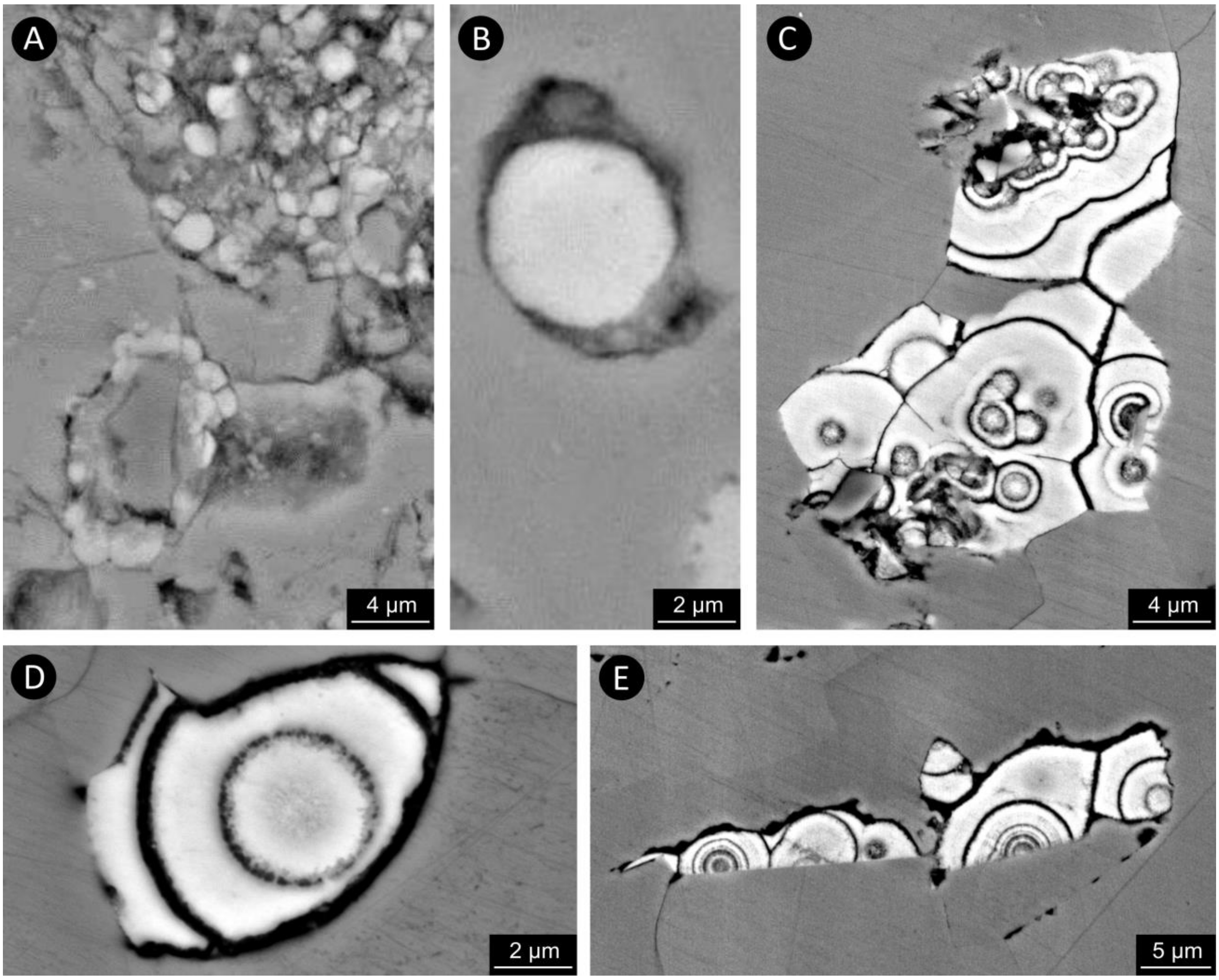

Scanning Electron Microscopy (SEM) images of spherulites

All images show spherulitic crystals likely consisting of calcium fluoride embedded in the 25.135 ± 0.537 ka to 23.524 ±0.446 ka layer in BV9b. A) Spherulites surrounding clasts in Dm. B) Spherulite preserved within Cs. C-E) Images in back scattered electrons (BSE) mode. The different shades indicate composition changes: calcite is light grey and calcium fluoride is white. Normalized weight % measured by EDX, and processed by Quantax software (Brucker Nano Gmbh) yielded F = 32.7%, Ca = 45.8%. Formation of calcium-fluoride spherulites suggests parent waters rich in F. Changes above threshold concentration of F in Antarctica ice cores have been related to volcanism[7]. Given that Boggs Valley is located within a tectono-magmatic region that has experienced active volcanism throughout the Quaternary[8,9], it is reasonable to infer that subglacial water bodies generated by volcanic eruptions were discharged down-glacier into Boggs Valley at peak glacial conditions.

Supplementary Figure 3

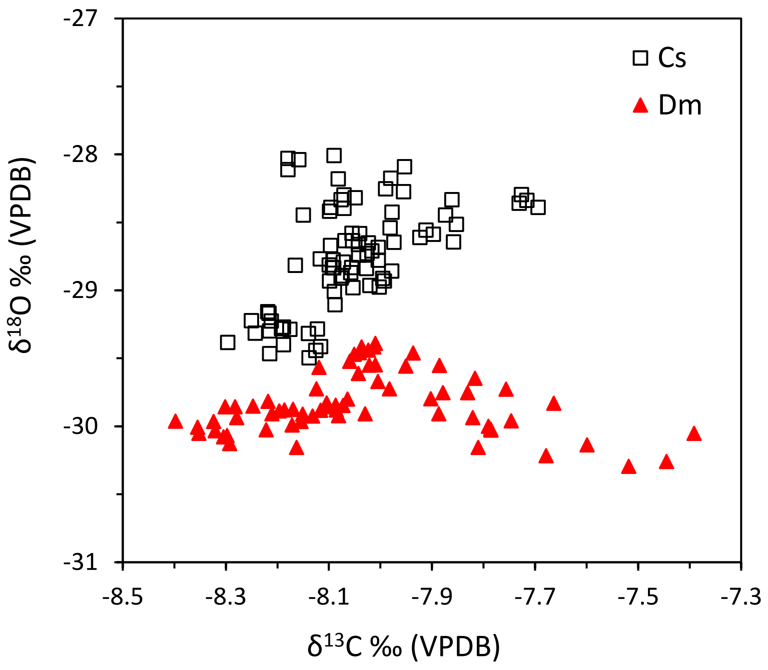

Stable isotope signals for Boggs Valley calcites

Stable C and O isotope ratios values distribution according to facies for BV9b; BV99a2, B9a2c and BV11. Negative $\delta^{18}O$ values for both Dm (red triangles) and Cs (open squares) are typical of other carbonates known to have precipitated from Antarctic glacial meltwaters[10]. The ~2 ‰ difference between $\delta^{18}O$ average values of Cs and Dm can be related to a different composition of basal ice melts[11]. Negative $\delta^{13}C$ of both Cs and Dm, as low as -8.4 ‰, suggests influence of microbial metabolism in subglacial environments[12]. Most commonly, calcium and carbonate ions are supplied to growing subglacial calcite surfaces via dissolution of a carbonate bedrock[13]. In the case of Boggs Valley, the bedrock is a non-carbonate and the supply of Ca can be ensured by bio-weathering of silicate minerals, whereas the CO_2 must be generated by microbial metabolism[14]. The hypothesis of bio-weathering of silicates in Boggs Valley as the provider of calcium and other trace elements that are incorporated in the calcite lattice, such as Fe (see text for details) is supported by exceptionally high U concentration (Supplementary Table 1). This is uncommon for continental calcites, and was likely to have been released by dissolution of silicates. The negative $\delta^{13}C$ values of the calcites suggests the presence of subglacial ecosystems with the characteristics of polar refugia dominated by microbial processes, leading to organic matter oxidation, remineralization of ^{12}C-enriched organic matter, sulphur oxidation, and possibly sulphate reduction[12].

Supplementary Figure 4

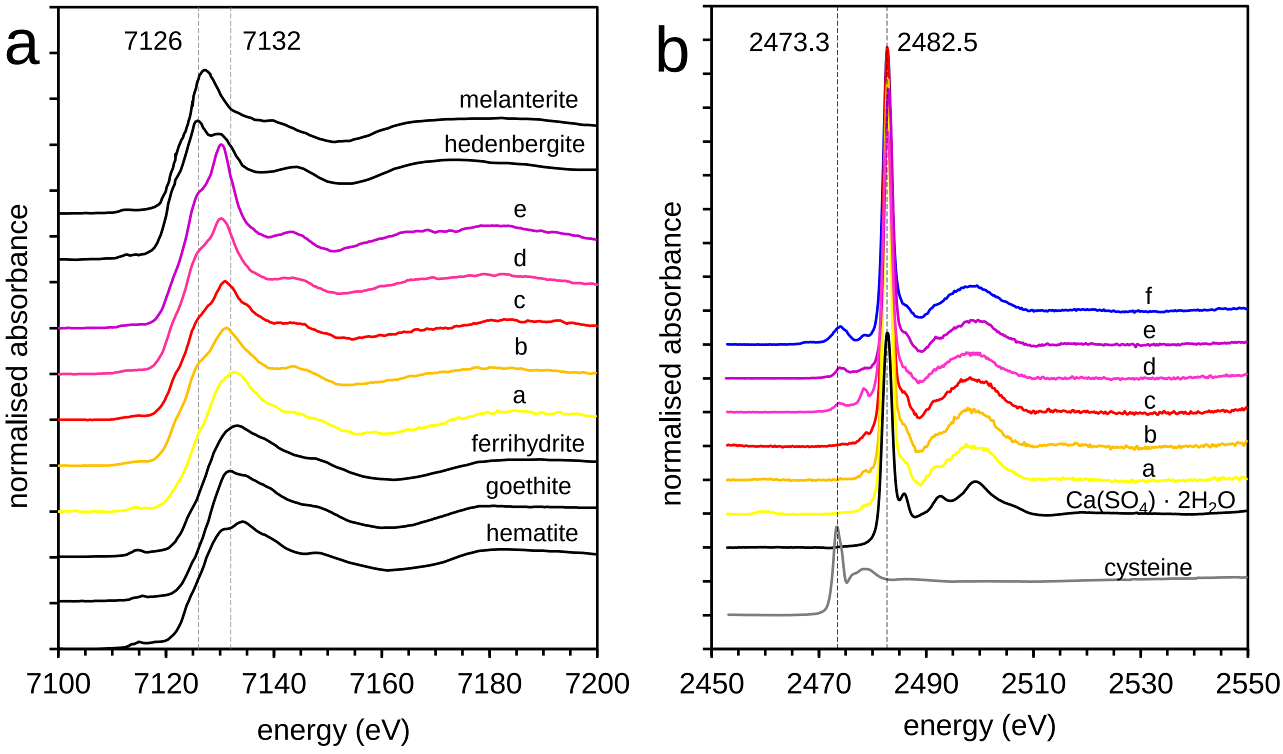

Micro X-ray Absorption Near Edge Structure (XANES) spectra of BV9b

a) Fe XANES spectra. The spectra were normalized for atomic absorption, based on the average absorption coefficient of the spectral region from 7200 to 7250 eV. A reference pure standard Fe foil (Kα edge at 7112 eV) was used to provide accurate calibration of the monochromator. Measured spectra on BV9b sample are those labelled from (a) to (e), the others are reference spectra. In XANES spectra (a) to (e) the strongest peak shows a progressive shift toward lower energies and a shoulder around 7126 eV. When compared with the references, this suggests that (a) spectrum corresponds to a species with $Fe^{(3+)}$ in 6-coordinated sites, as for ferrihydrite (best fitting spectra for point (a)), goethite and hematite (peak at 7132 eV). For (b) to (e) spectra, there is additional contribution of $Fe^{(2+)}$ in 6-coordinated sites as shown by the sharpest peak position of Fe^{2+} species such as hedenbergite ((Fe, Mg) $CaSi_2O_6$) and melanterite ($FeSO_4{\cdot}7H_2O$) (peaks at 7126 and 7130 eV).

b) Sulphur XANES spectra. The spectra were normalized on average absorption coefficient of the spectral region from 2510 to 2540 eV. A reference pure $Ca(SO_4){\cdot}2H_2O$ powder was used to provide an accurate calibration of the monochromator (maximum absorption at 2482.5 eV). All samples display a strong peak at 2482.5 eV characteristic of oxidised sulphate phases (ref. $Ca(SO_4){\cdot}2H_2O$). Samples (d) to (f) show a low intensity peak at 2473.3 attributable to organic compounds, possibly amino acids (ref. cysteine). The pre-edge peak at ~2478 eV is due to photo-reduction.

Supplementary Figure 5

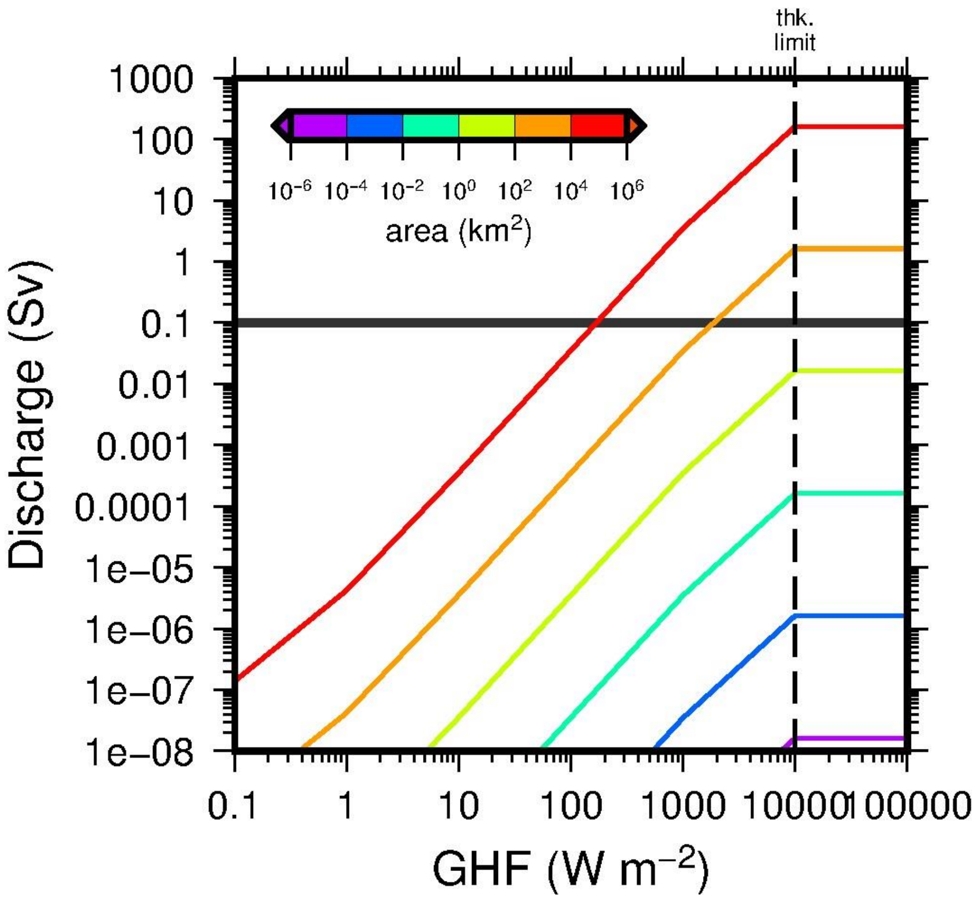

Estimated volumes of ice melt produced by subglacial volcanism

Discharges in Sverdrup (10^6 m^3 s^{-1}) estimated for subglacial eruptive volcanism for different areal extents and Geothermal Heat Fluxes (GHF) on the basis of Icelandic examples[15]. The GHF during the initial days of the subglacial eruption reached values six to seven orders of magnitude above the typical background rates[15]. Since the lateral extent of the elevated heat fluxes could not be constrained, a range of horizontal areas based on prescribed radii (r) from an arbitrary central point was used. An ice thickness (H) of 750 m calculated for the LGM (see main text), was used to limit meltwater production in the vertical dimension. By assuming radial reduction in ice melting away from the central volcanic source, total meltwater (M) was calculated as a conical volume (V) by using Equation S1:

$$V = \pi r^2 \frac{M}{3} \qquad \text{(S1)}$$

where $$M = \frac{G + \kappa_{ice} \frac{\partial T}{\partial z}}{\rho_i L_i} \qquad \text{(S2)}$$

The partial derivative in Equation S2 describes the vertical temperature gradient in the ice, which

was set to the conditions described in the text. The other terms are the ice density ρ_i and the latent heat of freezing L_i. The figure shows that volcanism elevates subglacial heat fluxes and dramatically increases the discharge if the melted ice area is in the range 10^2 to 10^6 km^2.
The horizontal black line in the figure denotes the approximate meltwater flux rate above which a significant impact on Antarctic Bottom Water formation would occur, based on climate modelling experiments[16]. The vertical dashed line (thk limit) simply means that any additional or ongoing heat will not melt any additional ice, because the ice that could be melted would already have done so.

The model implies the potential for rapid transfer of hydrothermally influenced waters from the plateau along the axis of the Boggs Valley and Rennick ice streams when the hydraulic barrier was breached.

Supplementary Figure 6

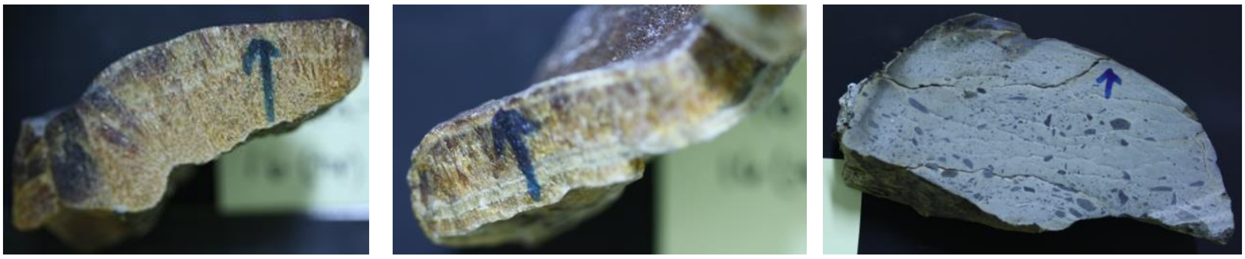

BV8a BV9b BV11

Hand specimens used for ancient DNA extraction

Sample BV8a and BV9b mostly consist of Cs and thin Dm layers and have similar stratigraphy. Sample BV11 is composed solely of Dm embedding angular to sub-angular sediment and particulate. Arrows show the top of the crusts.

Supplementary Table 1

Sample	Depth (mm)	Fabric	U (ppm)	[^{230}Th/ ^{238}U]	95% ext.	[^{234}U/ ^{238}U]	95% ext.	[^{232}Th/ ^{238}U]	95% ext.	[^{230}Th/ ^{232}Th]	Age (ka b2k)	2σ error (ka)	[^{234}U/ ^{238}U]i	95% ext.
BV8a-1	1.0	Cs		0.7342	±0.0183	4.7678	±0.0255	0.00150	±0.00008	491	17.805	±0.486	4.9619	±0.0263
BV8a-2	6.0	Cs		0.7880	±0.0126	4.7437	±0.0225	0.00059	±0.00009	1337	19.337	±0.347	4.9537	±0.0231
BV8a-3	10.0	Cs		0.8444	±0.0188	4.6958	±0.0255	0.00091	±0.00006	928	21.047	±0.521	4.9220	±0.0262
BV8a-4	17.0	Cs		0.8844	±0.0197	4.7227	±0.0313	0.00096	±0.00013	922	21.988	±0.553	4.9610	±0.0320
BV9a2	1.0	Cs	30.4 ±3.0	0.7205	±0.0128	4.8729	±0.0147	0.00139	±0.00001	519	17.054	±0.332	5.0639	±0.0153
BV9a2	2.0	Cs		0.7121	±0.0168	4.7436	±0.0320	0.00131	±0.00011	543	17.284	±0.449	4.9323	±0.0322
BV9a2	3.0	Cs		0.7263	±0.0082	4.8382	±0.0163	0.00233	±0.00001	312	17.299	±0.230	5.0303	±0.0167
BV9a2	5.0	Cs		0.7282	±0.0080	4.8094	±0.0116	0.00198	±0.00001	368	17.468	±0.219	5.0018	±0.0121
BV9a2	6.5	Cs		0.8047	±0.0101	4.8361	±0.0142	0.00095	±0.00002	849	19.359	±0.265	5.0516	±0.0146
BV9a2	7.5	Cs		0.8327	±0.0087	4.8150	±0.0170	0.00221	±0.00002	377	20.137	±0.249	5.0380	±0.0173
BV9a2	8.0	Cs		0.8140	±0.0091	4.7119	±0.0249	0.00134	±0.00002	607	20.144	±0.273	4.9290	±0.0253
BV9a2	9.0	Dm	58.9 ±3.9	0.8602	±0.0104	4.7691	±0.0124	0.00099	±0.00001	867	21.113	±0.283	5.0006	±0.0129
BV9a2	10.5	Cs		*0.9085*	*±0.0252*	*4.5764*	*±0.0237*	*0.05042*	*±0.00128*	*18*	*21.681*	*±1.883*	*4.8037*	*±0.0320*
BV9a2	12.0	Cs		0.9398	±0.0075	4.7735	±0.0228	0.00277	±0.00002	339	23.154	±0.252	5.0282	±0.0231
BV9a2	13.0	Cs		0.9920	±0.0078	4.8908	±0.0174	0.00162	±0.00001	612	23.954	±0.231	5.1630	±0.0177
BV9a2	14.0	Cs+Dm		1.0276	±0.0041	4.9315	±0.0142	0.00094	±0.00003	1098	24.634	±0.135	5.2152	±0.0145
BV9a2	14.5	Cs		1.0269	±0.0077	4.9277	±0.0130	0.00097	±0.00003	1061	24.635	±0.221	5.2106	±0.0131
BV9a2	15.5	Cs		*1.1299*	*±0.0358*	*4.6066*	*±0.0461*	*0.05545*	*±0.00095*	*20*	*27.641*	*±2.147*	*4.9008*	*±0.0520*
BV9a2a	1.0	Cs		0.9340	±0.0083	4.6535	±0.0147	0.00041	±0.00002	2305	23.661	±0.242	4.9072	±0.0152
BV9a2a	1.5	Cs+Dm		*1.0408*	*±0.0160*	*4.4240*	*±0.0290*	*0.09889*	*±0.00073*	*11*	*24.750*	*±3.621*	*4.6717*	*±0.0474*
BV9a2a	2.0	Cs+Dm		0.9203	±0.0154	4.6181	±0.0216	0.00107	±0.00005	859	23.524	±0.446	4.8664	±0.0223
BV9a2a	3.5	Cs+Dm	23.8 ±2.2	0.9636	±0.0122	4.4955	±0.0227	0.01091	±0.00009	88	25.135	±0.537	4.7524	±0.0234
BV9a2a	5.0	Cs		1.0049	±0.0222	4.5834	±0.0265	0.00234	±0.00011	429	26.070	±0.657	4.8569	±0.0277
BV9a2a	6.0	Cs		1.0585	±0.0110	4.5821	±0.0257	0.02197	±0.00015	48	26.951	±0.824	4.8651	±0.0272
BV9a2a	6.5	Dm		1.1035	±0.0098	4.6737	±0.0176	0.02290	±0.00039	48	27.534	±0.822	4.9724	±0.0203
BV9a2b	1.0	Cs		0.7232	±0.0083	4.6896	±0.0180	0.00074	±0.00002	972	17.803	±0.229	4.8810	±0.0184
BV9a2b	7.5	Cs		*0.8930*	*±0.0153*	*4.5540*	*±0.0200*	*0.05513*	*±0.00287*	*16*	*21.204*	*±1.940*	*4.7748*	*±0.0283*
BV9a2b	8.0	Dm		0.9437	±0.0104	4.6794	±0.0131	0.00083	±0.00005	1130	23.769	±0.296	4.9361	±0.0134
BV9a2b	10.0	Cs		*1.1238*	*±0.0098*	*4.5645*	*±0.0173*	*0.05075*	*±0.00075*	*22*	*27.910*	*±1.766*	*4.8586*	*±0.0258*
BV9a2c	0.5	Cs	24.8 ±1.6	0.7227	±0.0086	4.7324	±0.0216	0.00187	±0.00004	387	17.582	±0.241	4.9237	±0.0218
BV9a2c	1.0	Cs		0.7084	±0.0050	4.7571	±0.0349	0.00037	±0.00006	1893	17.216	±0.187	4.9441	±0.0350
BV9a2c	1.5	Cs		0.7993	±0.0099	4.6759	±0.0352	0.00068	±0.00002	1168	19.940	±0.312	4.8886	±0.0357
BV9a2c	2.5	Cs		0.8083	±0.0093	4.6741	±0.0357	0.00151	±0.00003	535	20.162	±0.303	4.8893	±0.0360
BV9a2c	3.5	Cs		0.8145	±0.0081	4.7300	±0.0277	0.00203	±0.00003	401	20.051	±0.258	4.9472	±0.0281
BV9a2c	4.5	Cs		0.8376	±0.0065	4.7500	±0.0202	0.00169	±0.00003	495	20.582	±0.203	4.9743	±0.0204
BV9a2c	10.0	Cc		0.8648	±0.0149	4.6869	±0.0198	0.00484	±0.00009	179	21.511	±0.443	4.9176	±0.0206
BV9a2c	14.0	Cc		0.9154	±0.0170	4.6986	±0.0251	0.01373	±0.00017	67	22.528	±0.661	4.9414	±0.0263
BV9b	0.5	Cs	41.6 ±3.5	0.7096	±0.0082	4.7332	±0.0142	0.00096	±0.00017	737	17.272	±0.226	4.9211	±0.0147
BV9b	4.0	Cs	43.1 ± 2.7	0.8169	±0.0161	4.7259	±0.0137	0.00012	±0.00008	6709	20.143	±0.431	4.9453	±0.0146
BV9b	9.0	Dm		0.9203	±0.0154	4.6181	±0.0216	0.00107	±0.00005	859	23.524	±0.446	4.8664	±0.0223
BV9b	12.5	Cs	35.7 ±1.7	1.0188	±0.0067	4.6073	±0.0119	0.00018	±0.00006	5667	26.307	±0.204	4.8871	±0.0122
BV9a1(i)	0.5	Cs	43.2 ± 0.5	0.7465	±0.0159	4.5307	±0.0222	0.02122	±0.00025	35	18.455	±0.867	4.7195	±0.0241
BV9a1(i)	1.0	Cs	55.7 ± 1.1	0.7969	±0.0163	4.6639	±0.0273	0.00295	±0.00007	270	19.856	±0.466	4.8751	±0.0281
BV9a1(i)	4.0	Cs	42.3 ± 0.4	0.8308	±0.0125	4.7371	±0.0421	0.00149	±0.00004	559	20.468	±0.389	4.9593	±0.0426
BV9a1(i)	6.0	Cc	38.8 ± 0.3	0.8605	±0.0257	4.6820	±0.0336	0.02068	±0.00019	42	20.891	±0.996	4.9056	±0.0356
BV9a1(i)	7.0	Cg	30.3 ±0.2	0.8739	±0.0255	4.7026	±0.0279	0.00574	±0.00012	152	21.649	±0.729	4.9358	±0.0292
BV9a1(i)	8.0	Cg	32.6 ±0.3	0.9186	±0.0083	4.6640	±0.0185	0.01127	±0.00009	82	22.882	±0.451	4.9085	±0.0192
BV9a1(i)	10.0	Cg	36.3 ±0.4	*1.2105*	*±0.0166*	*4.4784*	*±0.0279*	*0.17181*	*±0.00103*	*7*	*26.897*	*±6.275*	*4.7528*	*±0.0721*
BV11a(2)	0.5	Dm	45.1 ±2.4	0.8762	±0.0125	4.5779	±0.0170	0.02669	±0.00151	33	21.562	±0.981	4.8040	±0.0197
BV11a(2)	15.0	Dm	50.6 ±2.3	*0.9693*	*±0.0136*	*4.6435*	*±0.0128*	*0.03717*	*±0.00020*	*26*	*23.459*	*±1.283*	*4.8943*	*±0.0193*

U-series age data for Boggs Valley subglacial calcites.

"Depth (mm)" is the total extension from sample outer surface to its base. Fabrics codes are: Cs = clear, columnar calcite sparite; Cc = isopachous calcite cements; Cg = micrite coating grains (Coated grains); Dm = dirty microsparite. The U concentration is also reported where analysed. Activity ratios were determined using a Nu Plasma MC-ICP-MS at the University of Melbourne[17,18]. Ages are expressed in ka before year 2000 AD and were calculated by using the decay constants reported in ref.[19], corrected for initial ^{230}Th following the procedure proposed

by ref.[20] and by assuming an initial [^{230}Th/^{232}Th] of 1.5 ±1.5. "[^{234}U/^{238}U]i" is the reconstructed ^{234}U/^{238}U activity ratio at time of sample formation given its calculated age. Uncertainties are 95% external confidence intervals. The analyses reported in italics red have a [^{230}Th/^{232}Th] lower than 30, suggesting detrital contamination, and were excluded from the age distribution plot in Fig.4b.

Supplementary References

1 van Beynen, P., Bourbonniere, R., Ford, D. & Schwarcz, H. Causes of colour and fluorescence in speleothems. *Chemical Geology* **175**, 319-341 (2001).

2 De Yoreo, J. J. *et al.* Crystallization by particle attachment in synthetic, biogenic, and geologic environments. *Science* **349**, aaa6760 (2015).

3 Tessensohn, F. & Henjes-Kunst, F. Northern Victoria Land terranes, Antarctica: far-travelled or local products? *Geological Society, London, Special Publications* **246**, 275-291 (2005).

4 Dow, J. & Neall, V. Geology of the lower Rennick Glacier, northern Victoria Land, Antarctica. *New Zealand Journal of Geology and Geophysics* **17**, 659-714 (1974).

5 González-Muñoz, M. T., Chekroun, K. B., Aboud, A. B., Arias, J. M. & Rodriguez-Gallego, M. Bacterially induced Mg-calcite formation: role of Mg2+ in development of crystal morphology. *Journal of Sedimentary Research* **70** (2000).

6 Jones, B. Phosphatic precipitates associated with actinomycetes in speleothems from Grand Cayman, British West Indies. *Sedimentary Geology* **219**, 302-317 (2009).

7 Herron, M. M. Impurity sources of F^-, Cl^-, NO_3^- and SO_4^{2-} in Greenland and Antarctic precipitation. *Journal of Geophysical Research: Oceans* **87**, 3052-3060 (1982).

8 Kurbatov, A. V. *et al.* A 12,000 year record of explosive volcanism in the Siple Dome Ice Core, West Antarctica. *Journal of Geophysical Research: Atmospheres (1984–2012)* **111** (2006).

9 Ferraccioli, F. *et al.* Magmatic and tectonic patterns over the Northern Victoria Land sector of the Transantarctic Mountains from new aeromagnetic imaging. *Tectonophysics* **478**, 43-61 (2009).

10 Goodwin, I. D. & Hellstrom, J. Glacio-lacustrine aragonite deposition, meltwater evolution and glacial history during isotope stage 3 at Radok Lake, Amery Oasis, northern Prince Charles Mountains, East Antarctica. *Antarctic Science* **19**, 365-372 (2007).

11 Refsnider, K. A. *et al.* Subglacial carbonates constrain basal conditions and oxygen isotopic composition of the Laurentide Ice Sheet over Arctic Canada. *Geology* **40**, 135-138 (2012).

12 Neumann, K., Lyons, W. B., Priscu, J. C., Desmarais, D. J. & Welch, K. A. The carbon isotopic composition of dissolved inorganic carbon in perennially ice-covered Antarctic lakes: searching for a biogenic signature. *Annals of Glaciology* **39**, 518-524 (2004).

13 Sharp, M., Tison, J.-L. & Fierens, G. Geochemistry of subglacial calcites: implications for the hydrology of the basal water film. *Arctic and Alpine Research* **22**, 141-152 (1990).

14 Montross, S. N., Skidmore, M., Tranter, M., Kivimäki, A.-L. & Parkes, R. J. A microbial driver of chemical weathering in glaciated systems. *Geology* **41**, 215-218 (2013).

15 Gudmundsson, M. T., Sigmundsson, F., Björnsson, H. & Högnadóttir, T. The 1996 eruption at Gjálp, Vatnajökull ice cap, Iceland: efficiency of heat transfer, ice deformation and subglacial water pressure. *Bulletin of Volcanology* **66**, 46-65 (2004).

16 Menviel, L., Timmermann, A., Timm, O. E. & Mouchet, A. Climate and biogeochemical response to a rapid melting of the West Antarctic Ice Sheet during interglacials and implications for future climate. *Paleoceanography* **25**, PA4231 (2010).
17 Hellstrom, J. Rapid and accurate U/Th dating using parallel ion-counting multicollector ICP-MS. *Journal of Analytical Atomic Spectrometry* **18**, 1346-1351 (2003).
18 Drysdale, R. N. *et al.* Precise microsampling of poorly laminated speleothems for U-series dating. *Quaternary Geochronology* **14**, 38-47 (2012).
19 Cheng, H. *et al.* Improvements in ^{230}Th dating, ^{230}Th and 234U half-life values, and U-Th isotopic measurements by multi-collector inductively coupled plasma mass spectrometry. *Earth and Planetary Science Letters* **371-372**, 82-91 (2013).
20 Hellstrom, J. U–Th dating of speleothems with high initial ^{230}Th using stratigraphical constraint. *Quaternary Geochronology* **1**, 289-295 (2006).
